# Soft Multifunctional Porous Sponge Sensor for Pressure and Strain Using Liquid Metal/Polydimethylsiloxane with Silver-Nanowire-Coated Composite

**DOI:** 10.3390/mi13111998

**Published:** 2022-11-17

**Authors:** Dong-Young Kim, Kun-Woo Nam, Byung-Ho Kang, Sung-Hoon Park

**Affiliations:** Department of Mechanical Engineering, Soongsil University, 369 Sangdo-ro, Dongjak-Gu, Seoul 06978, Republic of Korea

**Keywords:** porous sponge, liquid metal, compression, tension

## Abstract

Compression and tension sensors with a porous structure have attracted attention recently. Porous sponge sensors have the advantage of a wide deformation range owing to their structural characteristics. In this study, a porous sponge structure was prepared by absorbing polydimethylsiloxane (PDMS) into the matrix of porous commercial sugar cubes. A conductive network was formed by coating the outside of the sponge skeleton with silver nanowires (AgNWs), which have a high aspect ratio. In addition, a liquid metal (LM), which does not directly form an electrical network but changes from zero-dimensional to one-dimensional under an external force was introduced into this porous sponge structure. The effects of the LM on the sensor sensitivity to pressure and strain were analyzed by comparing the electrical resistance changes of PDMS/AgNW and LM/PDMS/AgNW sponge sensors under tension and pressure. This study shows that the use of a porous structure and an LM may be useful for future wearable sensor design.

## 1. Introduction

Flexible and stretchable materials for robotic sensory skins and wearable devices are being studied because conventional sensors have intrinsic limitations under deformation. In sensors made of elastic materials, a conductive filler such as carbon nanotubes [1,2,3], carbon black [4,5], or silver nanowires (AgNWs) [6] is typically dispersed in a polymer material such as polyimide, polydimethylsiloxane (PDMS), or thermoplastic polyurethane [7,8,9].

AgNWs are nanoscale materials with excellent aspect ratios [10,11]. They are suitable for use as a conductive filler in composites. Because AgNWs are one-dimensional (1D), sensors containing them can sense the electrical resistance more easily under an external force such as tension or pressure. AgNWs form a conducting network when such forces are applied to a sensor. Even if the AgNWs are not physically in contact, the tunneling effect promotes electron transport between AgNWs to form an electrical pathway [12]. However, AgNWs are brittle and vulnerable to fracture, and thus their use in sensors is clearly limited.

Liquid metals (LMs), however, may be useful for supporting conductive nanofillers inside sensors. The LM, Galinstan, has the advantage of low toxicity to the human skin [13,14], which can be applied as a tension and pressure sensor in the movement of the finger [15]. Galinstan is liquid at room temperature but has metallic properties such as high thermal and electrical conductivity [16]. Because of their size and the oxide shells covering them, LMs are difficult to use as a single conducting filler at low strength, for example, a few kilopascals, which is typical of sensors [16,17,18]. However, because of their phase, they can deform freely under an external force from zero-dimensional (0D) to 1D [19].

Here, we fabricated a porous PDMS sponge sensor with an embedded LM and a AgNW coating. The porous structure is cost-effective, lightweight, and highly flexible [20,21]. By simply replicating the structural properties of commercial white sugar cubes using PDMS, we easily obtained a porous sponge body. Sugar cubes were soaked in PDMS and LM/PDMS solutions, and the sugar was removed after sponges formed. The sponge skeleton was coated with AgNWs to enhance its piezoresistivity. The performance of PDMS/AgNW and LM/PDMS/AgNW porous sponge sensors was compared, and the main function of the LM was experimentally demonstrated. These foam sensors have excellent potential for use as strain or pressure sensors in wearable devices, robotic fingers, and healthcare instruments [1,22,23,24].

## 2. Materials and Methods

### 2.1. Materials

The AgNWs used as a filler in the sponge sensor were purchased from DS Hi-Metal (Ulsan, Republic of Korea). Their average length was 20 μm, and the diameter was 40 nm. PDMS (Sylgard 184) from Dow Corning (Midland, MI, USA) was used as a matrix resin. The LM alloy (Galinstan, 61% Ga/25% In/13% Sn/1% Zn, by weight) used to enhance the electrical conductivity of the sensor was obtained from RND Korea (Gyeonggi, Republic of Korea). White sugar (Q.One, Seoul, Republic of Korea) was purchased from Samyang Corp. (Gyeonggi, Republic of Korea) to provide the porous structure of the sponge sensor. The dimensions of the sugar cubes were 15 × 15 × 15 mm^3^.

### 2.2. Fabrication of Silver-Nanowire-Coated Porous Sponge Sensor

**Figure 1:**
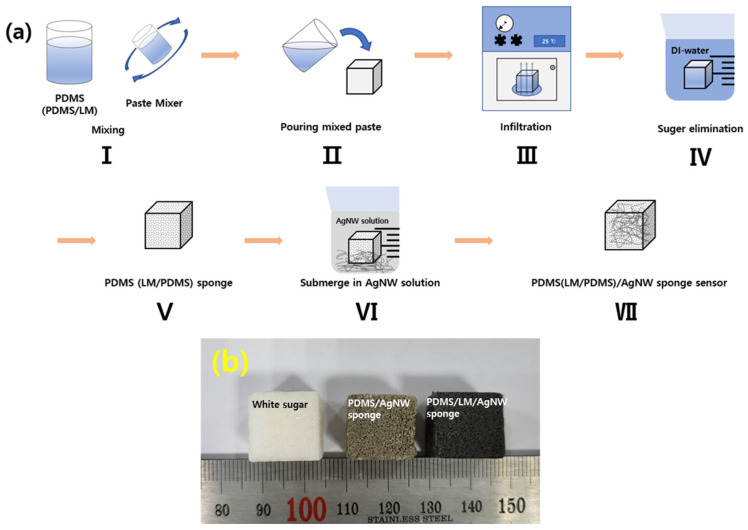
Soft multifunctional polydimethylsiloxane (PDMS)/silver nanowire (AgNW) and liquid metal (LM)/PDMS/AgNW porous sponge sensors. (**a**) Schematic illustration of fabrication of sponge sensor; (**b**) images of white sugar cube, PDMS/AgNW sponge, and PDMS/LM/AgNW sponge.

Commercial white sugar cubes were used to form the porous structure of the sponge sensor. To replicate the structure of white sugar cubes, low-viscosity PDMS and LM/PDMS solutions were poured over white sugar cubes. The fabrication of the sponge sensors is illustrated schematically in Figure 1a. In (step I), the PDMS and LM/PDMS solutions were evenly mixed using a paste mixer (Daehwa, Seoul, Republic of Korea) for 1.5 min. PDMS was prepared by mixing a prepolymer and PDMS curing agent (10:1 wt. ratio for PDMS prepolymer to curing agent), and the LM was added at a weight ratio of 50 wt.% relative to the mass of the PDMS. The mixed pastes were gently poured over the white sugar cubes to minimize air bubbles (step II), and a vacuum oven was used to allow the PDMS and LM/PDMS paste to thoroughly impregnate the sugar cubes. The mixtures were incubated overnight in a vacuum to ensure that paste reproduced the structure of sugar (step III). Next, each mixture was placed in deionized (DI) water at 80 °C for 1 h to solidify the polymers and remove the sugar (step IV). After all the sugar was removed, the sponges were held at room temperature for one day to evaporate residual DI water (step V). The sponge sensors were then submerged in an ethanol-based AgNW solution (step VI) to coat the outside of the sponge skeleton with AgNWs. The sponge was held in the solution for one day and then placed in an oven at 100 °C for 1 h to evaporate the solvent and stabilize the electrical conducting network. Finally, the dried samples were removed from the oven to obtain AgNW-coated PDMS and LM/PDMS porous sponge sensors (step VII). Figure 1b shows optical images of each sponge sensor and a sugar cube.

### 2.3. Measurement of Electrical Resistance under Applied Pressure or Tension

The electrical resistance of the PDMS/AgNW and PDMS/LM/AgNW sponge sensors was measured by a DMM 7510 digital multimeter (Keithley, Cleveland, OH, USA) using the two-wire method. As each electrode of the sponge sensor was connected to the multimeter, the sensor was loaded to a homemade XYZ three-dimensional stretching/pressing machine (Namil Optical Instruments Co., Incheon, Republic of Korea) that applied both pressure and tension. In tension mode, the machine stretched the sample at 12 mm/min. In load cell mode, the sample was stretched at 10 mm/min, and in cyclic mode it was stretched at 100 mm/min. The cyclic test was performed 500 times in a specific range to test the repeatability and durability of the sensor. The resistance recorded by the multimeter was plotted as the normalized resistance (R/R_0_), where R is the measured resistance, and R_0_ is the initial resistance.

## 3. Results and Discussion

### 3.1. Sample Morphology

**Figure 2:**
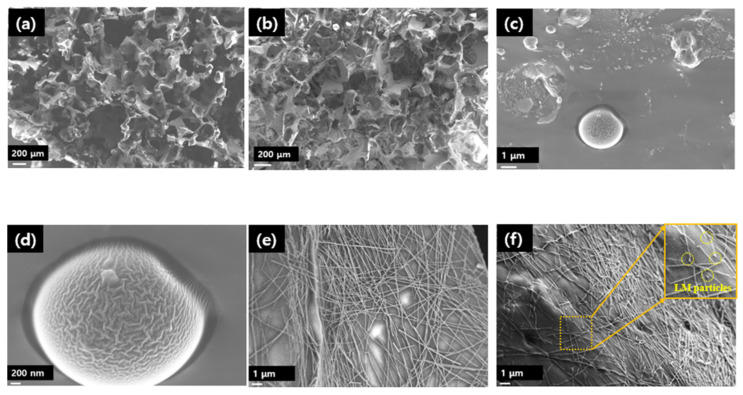
Cross-sectional SEM images of sponge sensors: structure of (**a**) PDMS/AgNW and (**b**) LM/PDMS/AgNW sensors. Images of LM at (**c**) low and (**d**) high magnification, and AgNWs in (**e**) PDMS/AgNW sponge and (**f**) LM/PDMS/AgNW sponge. Inset image indicates the LM particle.

Figure 2 depicts the morphology of the PDMS/AgNW and PDMS/LM/AgNW foams. The PDMS sponge shows a smooth surface and random pores that resemble the structure of a white sugar cube [25,26]. Both sponges were well made, as shown by the SEM images in Figure 2a,b. Low- and high-magnification SEM images (Figure 2c,d) show that the LM is distributed on the surface of the LM/PDMS/AgNW sponge, where it was well dispersed by the centrifugal force of the paste mixer. The LM, which is a liquid at room temperature, is separated into regions of various sizes by centrifugal force [17,27]. It promotes the formation of an electrical network by AgNWs in the sponge sensor, as described below. Figure 2e,f shows images of the AgNWs used as conductive fillers on the surfaces of the two types of sponges. The outside of the sponge skeleton was successfully coated with AgNWs simply by submerging it in the AgNW solution [25,28].

### 3.2. Electrical Resistance

#### 3.2.1. Tension Sensing Properties

**Figure 3:**
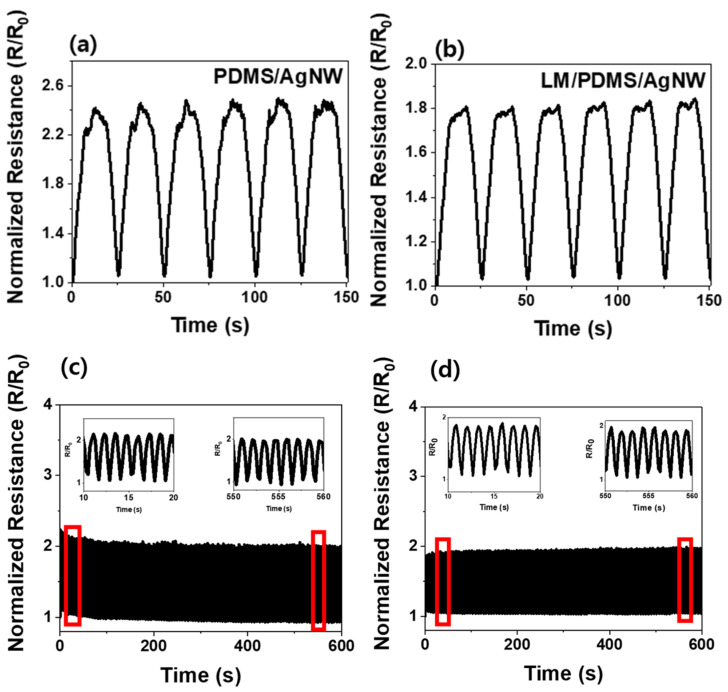
Normalized resistance versus time of PDMS/AgNW and LM/PDMS/AgNW under applied tension: low-speed cyclic test (six cycles) of (**a**) PDMS/AgNW and (**b**) LM/PDMS/AgNW sponge. High-speed cyclic test (500 cycles) of (**c**) PDMS/AgNW and (**d**) LM/PDMS/AgNW sponges. The insets show magnified views of regions outlined in red.

The electrical resistance of the sponge sensor was measured to determine the sensitivity of the sensor under an applied tensile force. The results are shown in Figure 3. The sponge sensor was strained by 50% of its initial size. The normalized resistance was measured as the sensor was stretched and restored. Figure 3a shows the electrical resistance of the PDMS/AgNW sponge sensor, which varies by up to 148% under tension compared to the initial resistance. Figure 3b shows the resistance of the LM/PDMS/AgNW sensor, which varies by up to 84% from the initial resistance. The value of the initial resistance of PDMS/AgNW and LM/PDMS/AgNW sponge sensor is 2 and 1 Ω, respectively. The sensor without the LM shows better resistance performance. The electrical path in these sensors was formed by AgNWs dispersed inside the sponge. When the electrical network is broken by tensile force, the degree of breakage with LM is less than that without LM. The reason is that 1D AgNWs and the 0D LM are mixed [19]. In addition, the LM, which is a liquid, changes to a 1D material as tension is applied [16,17,27,29]. Because tension sensors operate on the principle that the electrical resistance changes as the conductive network breaks [25,26], the PDMS/AgNW sponge sensor has better sensitivity.

Figure 3c,d shows the normalized resistance of the PDMS/AgNW and LM/PDMS/AgNW sensors, respectively, in cyclic tension tests. A tensile force of 30% was applied 500 times to test the repeatability and durability of the sensor [6,26]. The insets on the left and right show enlarged views of the beginning and end of the experiment, respectively.

#### 3.2.2. Pressure Sensing Properties

**Figure 4:**
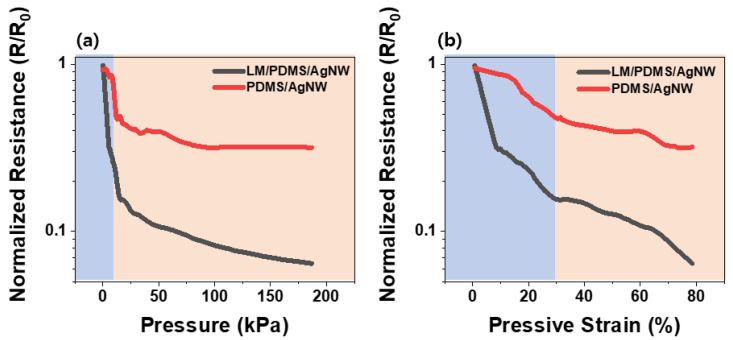
Normalized resistance of PDMS/AgNW (red) and LM/PDMS/AgNW (black) sponge sensors. (**a**) Normalized resistance versus pressure under applied pressure and (**b**) normalized resistance versus compressive strain under applied pressure. Blue region: contact zone. Orange region: piezoresistive zone.

**Figure 5:**
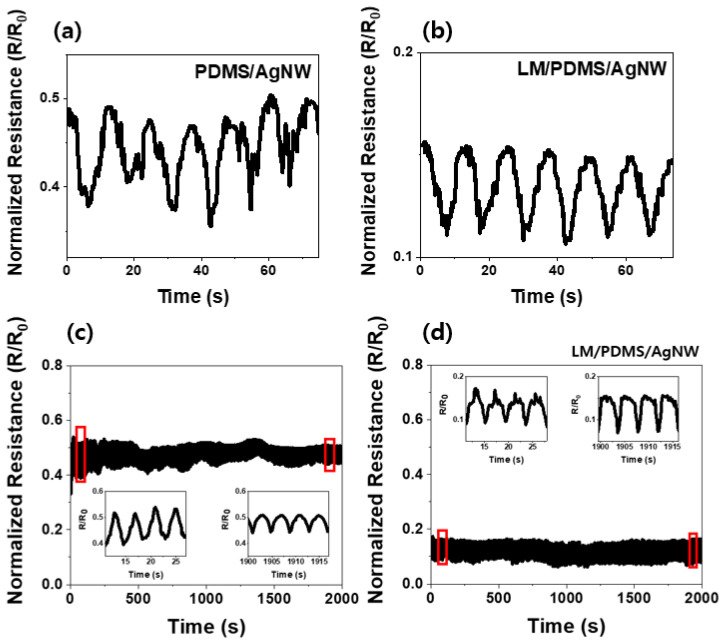
Normalized resistance versus time of PDMS/AgNW and LM/PDMS/AgNW sensors under applied pressure: low-speed cyclic test (six cycles) of (**a**) PDMS/AgNW and (**b**) LM/PDMS/AgNW sponges. High-speed cyclic test (500 cycles) of (**c**) PDMS/AgNW and (**d**) LM/PDMS/AgNW sponges. The insets show magnified views the regions outlined in red.

In tension mode, the LM did not improve the sensor performance. Rather, it degraded the sensitivity, because the fillers (AgNWs) inside the sensor were cut off with increasing strain [28]. However, in compression mode, the LM offers an advantage. Figure 4a shows the real-time resistance of the two types of sensors in terms of the normalized resistance. Because the sensor is inherently porous, the conductive fillers inside the sensor have few opportunities to come into contact when a force is applied. Additionally, because of the voids in the sponge, the deformation rate is high even under low pressure. In the low-pressure range (blue region), the resistance of the sponge decreases sharply as the sponge skeleton comes into direct contact with the conductive fillers on its surface. The resistance variates up to 53% and 84.8% of the PDMS/AgNW and LM/PDMS/AgNW sensor, respectively. As the pressure gradually increases, the resistance starts to decrease relatively slowly (orange region). This section shows piezoresistivity without structural characteristics [15,30]. Pressure was also applied at a compressive strain rate of 80%. The electrical resistance of the PDMS/AgNW and LM/PDMS/AgNW sensor changes up to 94% and 69%, respectively. Figure 4b shows the normalized resistance versus compressive strain. The PDMS/AgNW and LM/PDMS/AgNW sponges begin to saturate at approximately 13 and 20 kPa, respectively, at which the compressive strain is 30%. Figure 5 illustrates the benefits of adding LM to the sensor. A cyclic test was performed at a compressive strain of 30–60%, at which piezoresistivity appeared. Because the compressive strain was varied, the force applied to each sponge was slightly different. Therefore, in Figure 5, the sensor signal observed in repeated tests was compared rather than the change in the resistance of each sensor. The results demonstrate the durability and repeatability of the sensor. The initial resistance (*R*_0_) in this figure is the same as that obtained in Figure 4. Figure 5a,b shows the results of six cycles in the given pressure range. The PDMS/AgNW sensor exhibited highly unstable performance; by contrast, the LM/PDMS/AgNW sensor was relatively stable. Figure 5c,d shows the results of 500 cycles in the same pressure range. The insets show enlarged views of the beginning and end of the experiment. The resistance signal of the PDMS/AgNW sensor (Figure 5c) is unstable. In addition, the range in which the resistance changes decreased slowly (insets). The reason is that repeated pressure damages the AgNWs coated on the sponge skeleton. Because the AgNWs are brittle, the damage makes it difficult to form an electrical network on the sponge as the length is decreased by damage. The disruption of the network results in unstable resistance and degraded performance [28]. By contrast, Figure 5d shows relatively stable behavior compared to that in Figure 5c. The length of the LM increases as it changes from 0D to 1D under pressure. Because the shape of the AgNWs does not change significantly under pressure, the LM helps the nanowires to form an electrical pathway, and because the LM is liquid, it withstands pressure. Bulk force is required to damage the LM, and in the cyclic tests performed here (Figure 5), the damage to the LM could be very minor [16]. In conclusion, the sponge sensor was stable, and a comparison of the resistance at the beginning and end of the experiment reveals that the resistance range did not change significantly. However, damage to the AgNWs appeared even in the LM/PDMS/AgNW sponge, as indicated by the peaks at the beginning and end of the test.

### 3.3. Mechanism of Sponge Sensor

**Figure 6:**
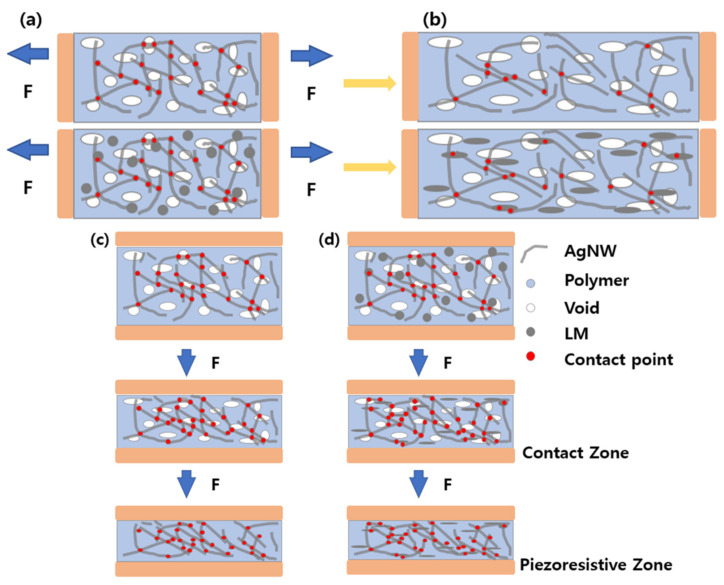
Schematic illustration of both types of sponge sensors. Internal view under applied tension of (**a**) PDMS/AgNW; (**b**) LM/PDMS/AgNW sensor and applied pressure of (**c**) PDMS/AgNW; (**d**) LM/PDMS/AgNW sensor.

The resistance of the sensor under tension and pressure has been demonstrated. Figure 6 shows a schematic illustration of its mechanism. Figure 6a,b shows the interior of the sponge under a tensile force. In the PDMS/AgNW sensor (Figure 6a), there are 19 contact points in the initial state. As tension is applied, the number of contact points decreases to 11. This result illustrates that the resistance increases as the number of contact points decreases, as in a composite sensor with a general conductive filler. However, in the LM/PDMS/AgNW sponge (Figure 6b), the number of contact points decreases from 19 to 16 as the tensile force increases. Although the initial state is similar to that of the sensor without the LM, as the sensor is stretched, the length of the LM increases as well, allowing it to behave as a 1D material instead of a 0D material [17]. Because the LM possesses electrical conductivity, it acts as a bridge between AgNWs, further reducing the loss of electrical connections because of tension, and the sensitivity decreases when LM is added to the sponge.

By contrast, under pressure, the LM instead improves the electrical pathway. Figure 6c,d shows the interior of both types of sponge under compression. For the PDMS/AgNW sponge (Figure 6c), there are initially 23 contact points, and the number of contact points increases to 29 when pressure is applied. As in Figure 6a, this result is similar to that of a composite compression sensor with a general conductive filler [30]. In the sensor with LM (Figure 6d), as in Figure 6b, the LM changes from 0D to 1D because of compression. More notably, the physical distance between the conductive fillers decreases under compression compared to that in tension mode, making it easier to form a conductive network. Moreover, the LM forms an additional electrical path by acting as a bridge between AgNWs at distances that are difficult to cover without the LM. The number of contact points increases from 23 to 34.

## 4. Conclusions

In summary, an LM/PDMS/AgNW sponge sensor modeled after the porous structure of sugar cubes was developed. PDMS is a representative polymer material with great flexibility, and by fabricating the sensor with a porous structure, creates better flexibility. The durability of the sensor was reinforced by using material and structural properties. The sensor can reliably detect external forces, specifically tension and pressure. The resistance performance of the sensor in 50% tension, PDMS/AgNW and LM/PDMS/AgNW sensor’s electrical resistance varies up to 148% and 84%, respectively. The durability and repeatability of the sensor were shown by the cyclic test of 500 times in 30% strain. In compressive mode, the PDMS/AgNW and LM/PDMS/AgNW sensor shows the resistance change up to 69% and 94%, respectively. A cyclic test was also applied in the pressing mode to show the repeatability and durability of the sensor. For each external force, we discussed the effects of the LM on the stability of the sensor. Although the addition of LM does not offer a significant advantage in tension mode, it enhances the sensitivity of the sensor in pressure mode even when the conductive silver nanowires were damaged. The scaffold guarantees durability for the porous structure and material properties, and the LM supports the role of AgNW, an internal conductive filler, suggesting the possibility of extending the lifespan of the overall sensor. This multifunctional sensor is expected to be useful in wearable devices. Because the LM is harmless to human skin, it can be applied as a sensor where soft external forces are needed such as the movement of the human finger or soft robotic grippers.

## References

[1] Zhai T., Li D., Fei G., Xia H. (2015). Piezoresistive and compression resistance relaxation behavior of water blown carbon nanotube/polyurethane composite foam. Compos. Part A Appl. Sci. Manuf..

[2] Kang B.-H., Jeong I.-Y., Park S.-H. (2022). Design of a Smart Conducting Nanocomposite with an Extended Strain Sensing Range by Conjugating Hybrid Structures. Polymers.

[3] Xie X., Ye M., Hu L., Liu N., McDonough J.R., Chen W., Alshareef H.N., Criddle C.S., Cui Y. (2012). Carbon nanotube-coated macroporous sponge for microbial fuel cell electrodes. Energy Environ. Sci..

[4] Yoo J., Kim D.-Y., Kim H., Hur O.-N., Park S.-H. (2022). Comparison of Pressure Sensing Properties of Carbon Nanotubes and Carbon Black Polymer Composites. Materials.

[5] Zhai W., Xia Q., Zhou K., Yue X., Ren M., Zheng G., Dai K., Liu C., Shen C. (2019). Multifunctional flexible carbon black/polydimethylsiloxane piezoresistive sensor with ultrahigh linear range, excellent durability and oil/water separation capability. Chem. Eng. J..

[6] Zhang S., Liu H., Yang S., Shi X., Zhang D., Shan C., Mi L., Liu C., Shen C., Guo Z. (2019). Ultrasensitive and highly compressible piezoresistive sensor based on polyurethane sponge coated with a cracked cellulose nanofibril/silver nanowire layer. ACS Appl. Mater. Interfaces.

[7] Ma H., Yang Y. (2008). Rheology, morphology and mechanical properties of compatibilized poly (vinylidene fluoride)(PVDF)/thermoplastic polyurethane (TPU) blends. Polym. Test..

[8] Jiang X., Bin Y., Matsuo M. (2005). Electrical and mechanical properties of polyimide–carbon nanotubes composites fabricated by in situ polymerization. Polymer.

[9] Lee J., Kim J., Shin Y., Jung I. (2019). Ultra-robust wide-range pressure sensor with fast response based on polyurethane foam doubly coated with conformal silicone rubber and CNT/TPU nanocomposites islands. Compos. Part B Eng..

[10] Kim J.M., Jang K.S., Lee S.J. (2019). Electrically conductive polystyrene nanocomposites incorporated with aspect ratio-controlled silver nanowires. J. Appl. Polym. Sci..

[11] Huang H., Cai C.J., Yeow B.S., Ouyang J., Ren H. (2021). Highly Stretchable and Kirigami-Structured Strain Sensors with Long Silver Nanowires of High Aspect Ratio. Machines.

[12] Du F., Scogna R.C., Zhou W., Brand S., Fischer J.E., Winey K.I. (2004). Nanotube networks in polymer nanocomposites: Rheology and electrical conductivity. Macromolecules.

[13] Chiechi R.C., Weiss E.A., Dickey M.D., Whitesides G.M. (2008). Eutectic gallium–indium (EGaIn): A moldable liquid metal for electrical characterization of self-assembled monolayers. Angew. Chem. Int. Ed..

[14] Dickey M.D., Chiechi R.C., Larsen R.J., Weiss E.A., Weitz D.A., Whitesides G.M. (2008). Eutectic gallium-indium (EGaIn): A liquid metal alloy for the formation of stable structures in microchannels at room temperature. Adv. Funct. Mater..

[15] Guo Z., Mo L., Ding Y., Zhang Q., Meng X., Wu Z., Chen Y., Cao M., Wang W., Li L. (2019). Printed and flexible capacitive pressure sensor with carbon nanotubes based composite dielectric layer. Micromachines.

[16] Liu Y., Ji X., Liang J. (2021). Rupture stress of liquid metal nanoparticles and their applications in stretchable conductors and dielectrics. Npj Flex. Electron..

[17] Bartlett M.D., Kazem N., Powell-Palm M.J., Huang X., Sun W., Malen J.A., Majidi C. (2017). High thermal conductivity in soft elastomers with elongated liquid metal inclusions. Proc. Natl. Acad. Sci. USA.

[18] Fassler A., Majidi C. (2015). Liquid-phase metal inclusions for a conductive polymer composite. Adv. Mater..

[19] Li Y., Cui Y., Zhang M., Li X., Li R., Si W., Sun Q., Yu L., Huang C. (2022). Ultrasensitive Pressure Sensor Sponge Using Liquid Metal Modulated Nitrogen-Doped Graphene Nanosheets. Nano Lett..

[20] Michel T.R., Capasso M.J., Cavusoglu M.E., Decker J., Zeppilli D., Zhu C., Bakrania S., Kadlowec J.A., Xue W. (2020). Evaluation of porous polydimethylsiloxane/carbon nanotubes (PDMS/CNTs) nanocomposites as piezoresistive sensor materials. Microsyst. Technol..

[21] Park J., Lee Y., Barbee M.H., Cho S., Cho S., Shanker R., Kim J., Myoung J., Kim M.P., Baig C. (2019). A hierarchical nanoparticle-in-micropore architecture for enhanced mechanosensitivity and stretchability in mechanochromic electronic skins. Adv. Mater..

[22] Zhang L., He J., Liao Y., Zeng X., Qiu N., Liang Y., Xiao P., Chen T. (2019). A self-protective, reproducible textile sensor with high performance towards human–machine interactions. J. Mater. Chem. A.

[23] Song Y., Chen H., Su Z., Chen X., Miao L., Zhang J., Cheng X., Zhang H. (2017). Highly compressible integrated supercapacitor–piezoresistance-sensor system with CNT–PDMS sponge for health monitoring. Small.

[24] Dong X., Wei Y., Chen S., Lin Y., Liu L., Li J. (2018). A linear and large-range pressure sensor based on a graphene/silver nanowires nanobiocomposites network and a hierarchical structural sponge. Compos. Sci. Technol..

[25] Lo L.-W., Zhao J., Wan H., Wang Y., Chakrabartty S., Wang C. (2022). A Soft Sponge Sensor for Multimodal Sensing and Distinguishing of Pressure, Strain, and Temperature. ACS Appl. Mater. Interfaces.

[26] Iglio R., Mariani S., Robbiano V., Strambini L., Barillaro G. (2018). Flexible polydimethylsiloxane foams decorated with multiwalled carbon nanotubes enable unprecedented detection of ultralow strain and pressure coupled with a large working range. ACS Appl. Mater. Interfaces.

[27] Yu D., Liao Y., Song Y., Wang S., Wan H., Zeng Y., Yin T., Yang W., He Z. (2020). A Super-Stretchable Liquid Metal Foamed Elastomer for Tunable Control of Electromagnetic Waves and Thermal Transport. Adv. Sci..

[28] Peng S., Wu S., Yu Y., Xia B., Lovell N.H., Wang C.H. (2020). Multimodal capacitive and piezoresistive sensor for simultaneous measurement of multiple forces. ACS Appl. Mater. Interfaces.

[29] Bartlett M.D., Fassler A., Kazem N., Markvicka E.J., Mandal P., Majidi C. (2016). Stretchable, high-k dielectric elastomers through liquid-metal inclusions. Adv. Mater..

[30] Meng Q., Lu Y., Wang J., Chen D., Chen J. (2021). A piezoresistive pressure sensor with optimized positions and thickness of piezoresistors. Micromachines.

